# Genetic characterization of group A rotavirus in children with acute gastroenteritis in Kericho County Referral Hospital, Kenya

**DOI:** 10.11604/pamj.2024.47.197.40761

**Published:** 2024-04-18

**Authors:** Beth Khayeli Nyamanga, Janeth Kombich, Carlene Sang, James Nyangao, Raphael Lihana

**Affiliations:** 1 School of Science and Technology, University of Kabianga, Kericho, Kenya; 2 Centre for Virus Research, Kenya Medical Research Institute (KEMRI), Nairobi, Kenya

**Keywords:** Electropherotypes, gastroenteritis, rotavirus, genotype

## Abstract

**Introduction:**

approximately over 80% of mortalities due to rotavirus occur in countries that have limited resources, especially in sub-Saharan Africa and South Asia. The study was intended to determine the genetic characteristics of rotavirus A in children exhibiting gastroenteritis at Kericho County Referral Hospital.

**Methods:**

the study design was cross-sectional. Consecutive sampling was engaged obtaining a sample size of 200 stool samples. Genetic characterization of group A rotavirus strains was done using Enzyme-Linked Immunosorbent Assay. Positive samples underwent Sodium dodecyl sulfate-polyacrylamide gel electrophoresis. Afterwards viewing of the RNA double strands of the rotavirus genome in gels was done using Silver Nitrate. The positive samples underwent RT-PCR amplification followed by sequencing on the pieces of the VP7 or VP4 gene obtained.

**Results:**

one hundred and six (53%) samples from males and 94 (47%) from females. Twenty-three samples were positive hence a prevalence of 11.5%. The most affected demographics were children of guardians with secondary school education (51%). The most affected social economic status was housewives (46.5%). The most affected age was 21-30 months at 26.5%. Long electropherotypes were in 22 samples (96%). The G3 genotype of rotavirus A was prevalent 16/23 (69.57%).

**Conclusion:**

rotavirus prevalence was 11.5%. The G3 genotype was the most prevalent in circulation. The occurrence of non-typable strains indicated that the strains may be diversified emphasizing the need to include emerging strains within the vaccines in use. Hence the need to continuously monitor the effects in older children.

## Introduction

Acute gastroenteritis amongst children below 5 years is often attributed to rotavirus infections [1]. A rotavirus particle is comprised of a capsid enclosing a dual-stranded ribonucleic acid (dsRNA), a characteristic found in the family Reoviridae [2]. Rotavirus ribonucleic acid (RNA) comprises 11 gene segments bounded by three concentric shells. Rotavirus infections are at least experienced once in most children around the world before or at the age of five years old. As infections keep recurring, immunity develops thus adults are least affected. Between 1996 and 2005 five strains of rotavirus “A” accounted for 90% of infections in children five years and below, that is genotype G1- G4 and G9 in the US. As of 2016 acute gastroenteritis due to rotavirus resulted in the death of 128500 youngsters under the age of 5 years of which over 100,000 happened in sub-Saharan Africa [3]. Group A of rotavirus contributes to more than 90% of infections thus making it the most prevalent in humans. Rotavirus infections of Group B strains are rare but associated with infections in Asia and China [4]. Children in Kenya have exhibited Group A, B, and C species of rotavirus strains [5]. It has been shown that there is a great variety of strains of rotavirus present in Africa [6]. Rotavirus is stable and remains viable in a locality indefinitely if the area is not disinfected. It is spread through the oral-fecal pathway. The rotavirus particles invade and destroy cells of the epithelial layer that lines the small intestine resulting in gastroenteritis [7]. The World Health Organization Strategic Advisory Group of Experts recommended the assimilation of the rotavirus vaccine into the vaccination programs for youngsters aged five years and under in 2009 [8]. In Kenya, this vaccination was introduced in 2014. Rotarix Vaccine was Kenya´s vaccine of choice administered in all public health facilities twice, at eight weeks and 16 weeks.

Globally, there is a general reduction in the number of hospitalizations more so, in first-world countries that experience fewer infant mortalities due to their effective immunization programs, compared to third-world countries that have higher infant mortalities [9]. In Kenya, a 15.2% prevalence had previously been observed confirming a higher prevalence within the age range of 13-24 months during the cold and wet months [10]. On the contrary some areas experience ineffective vaccination owing to the socio-economic, environmental and cultural beliefs. This has led to great variations in existing and re-emerging rotavirus strains particularly, in children who had already been vaccinated [11]. This raises concern on whether the strain in the Rotarix vaccine which is a single rotavirus variant of live weakened human 89-12 (type G1P1A [8]) evokes an efficient broad defense, against new strains that were not part of the vaccine as currently constituted. Hence there is a necessity for constant investigation of rotavirus strains so as to guide the efficacy of the vaccines in use. The effects of vaccine introduction in Kenya have been documented [12], but not to the extent of including its effect on the existing strains, demographics, and socio-economic aspects of agricultural communities especially one such as in Kericho County. Hence monitoring of circulating strains is necessary to guide the efficacy of vaccines in use. The county experiences extreme cold and wet climate which has been attributed to high transmission of infectious diseases.

Despite the inclusion of Rotarix Vaccine in the National Vaccination Program there are bound to be some effects on the levels of morbidity, mortality, and the types of strains existing in a population after vaccine introduction. This might be due to environmental influences in addition to several other factors. There is seasonality in rotavirus infections in Kenya. These infections increase in frequency such as between January to March as well as from June to September [10]. Rotavirus strains are bound to also be affected since most viruses tend to evolve in a way that aims to enable them to evade the immune system. Most rotavirus infections are normally not clinically diagnosed since the signs and symptoms are similar to other gastrointestinal infections. Vaccine introduction effectiveness is also affected by other population dynamics. This also includes the existence of other health conditions, socio-economic factors, education backgrounds, sanitation and environmental factors. The general objective of the study was to determine the prevalence and genetic diversity of group A rotavirus in children with acute gastroenteritis attending Kericho County Referral Hospital. The specific objectives were to determine the prevalence of gastroenteritis caused by rotavirus in children of five years and below in Kericho County Referral Hospital.

## Methods

**The study design:** this was a hospital-based cross-sectional study. The study site was Kericho County Referral Hospital in Kericho County South of Rift Valley.

**Participant selection:** the study participants consisted of children aged five years and below, visiting the paediatric clinic, exhibiting symptoms of acute gastroenteritis at the hospital. They were selected using consecutive sampling until the required sample size was reached. Vaccination against rotavirus was confirmed by the immunization booklet or word of mouth from parent/guardian. The consent was obtained from the guardians in written form with an outline of the process the samples would undergo for the research.

**Sample size estimation:** sample size determination was done using the formula by Charan & Biswas, 2013 [13]. A 95% confidence interval set at 1.96 with an estimated prevalence of rotavirus infections among children in Kenya of 14.5% as calculated and reported by Muendo *et al*. 2018 [14]. The final sample size obtained was 200 participants.

**Sample collection:** stool samples were collected from patients presenting with symptoms of gastroenteritis. They were frozen at -20°C awaiting for transportation in ice packed cold boxes to Kenya Medical Research Institute. Laboratory work was carried out in Biosafety level 2 in line with the WHO Biosafety manual. The inclusion criterion was children below 5 years showing symptoms of acute gastroenteritis. Children with bloody diarrhea were excluded.

**Specimen processing:** a small amount (roughly 10gm) of solid fecal matter or 700µl of loose fecal aliquots were collected in cryovials and put in storage at low temperatures of -20°C pending analysis. The fecal aliquots obtained from patients with symptoms of gastroenteritis were used to prepare 1ml of 10% (w/v) fecal suspension in phosphate-buffered saline (PBS), balanced salt solution (BSS), M199, or 0.01M Tris solution (pH 7.5, 14.5mM NaCl, 10mM CaCl2). This was vortexed and clarified through centrifugation. The supernatant formed was collected and stored at -70°C for long term [15]. Rotavirus Enzyme-Linked Immunosorbent Assay Kit was used to confirm Group A rotavirus antigens in the collected samples. The procedure was carried out as per the instructions in the WHO manual for Rotavirus detection and characterization. The results were read spectrophotometrically at 450nm. The Negative Control value was less than 0.150 absorbance units. The Positive Control value was greater than 0.500 absorbance units [15].

The positive samples further underwent Sodium dodecyl sulfate-polyacrylamide gel electrophoresis (SDS PAGE). It first entails the extraction of Rotavirus RNA from the fecal suspension through the use of Phenol- Chloroform. This process was then followed by Silver Staining of the double strand RNA in gels through the use of Silver Nitrate which assisted in viewing the RNA bands of the rotavirus genome. This procedure confirmed whether the strains obtained were either long or short electropherotypes [15]. The remaining fecal aliquots of positive samples were used to do RT-PCR amplification by first extracting RNA using QUIAGEN. This was followed by RT-PCR for G genotyping as per the World Health Organization protocols made available by the West African Regional Rotavirus Laboratory. RT-PCR of rotavirus dsRNA included: dsRNA denaturation, Reverse transcription, and amplification of the newly formed cDNA. Consensus primers sBeg 9 (GGCTTTAAAAGAGAGAATTTC)- position 1-21 and End 9 (GGTCACATCATACAATTCTAATCTAAG) - position 1062-1036 for rotaviruses that belong to group A [16]. sBeg and End 9 will be used in the first cycles of RT-PCR amplification. In RT-PCR for P Genotyping, VP4 genotype amplification was carried out using consensus primer con3 and a mixture of specific primers 1T-1, 2T-1, 3T-1, 4T-1 and 5T-1 to identify rotavirus VP4 genotypes [17]. Positive and negative controls were included.

The PCR fragments were run on 2% TAE agarose gel at 100 volts with suitable molecular weight markers to define the genotype of the rotavirus strain [15]. In verification, sequencing was done on genotype-specific yields or a piece of the VP7 or VP4 gene once amplification was completed. The ability to check for infections through purification and sequencing of products of diverse sizes sequestered from agarose gel was an added advantage of sequencing genotype-specific PCR yields [18]. As for the VP7 gene, a range of consensus primer pairs were defined together with beg9/end9 and VP7-F/VP7-R and degenerate versions, 9con1/9con2, and 9con1L/VP7-R deg. Consensus primers for VP4 gene fragments included con2/con3, Hum Com5/Hum Com3, and VP4-F/VP4-R. The PCR conditions consisted of denaturation which took place at 95°C for a duration of five minutes and this included 40 cycles, next was denaturation at 94°C for a minute and annealing at 55°C for a duration of one minute. The last step was elongation at 72°C for one minute, and afterward a last extension step at 72°C for ten minutes. Once sequencing is done, the strain genotypes are identified by matching the genes of strains recognized as VP4 or VP7 types by the Gen Bank database 40.

### Data analysis

The circulating strains of rotavirus were characterized and the results were compared to previous data collected from the populations before the rotavirus vaccine was introduced. Determination of demographic characteristics of children below five years presenting with rotavirus-related gastroenteritis was done by finding the percentage of the infected cases among the different ages. This was compared to previous studies carried out in Kenya on the same to enable finding out if there is a shift in the ages that normally experience rotavirus infections. This enabled the study to pinpoint the ages vulnerable to infections due to rotavirus [19]. In the determination of the occurrence of rotavirus-related gastroenteritis among children below five years, a percentage was calculated from the number of rotavirus-positive samples against the sum total of fecal samples collected during the study. This was compared to previously done hospital cross-sectional studies in other hospitals. Determination of genetic diversity of circulating strains of rotavirus among children five years and below, RNA extraction, Reverse Transcription, G and P-typing of Rotaviruses was done followed by sequencing of the VP7 and VP4 gene of the rotavirus positive strains. This was later followed by sequence and phylogenetic analysis based on VP4 and VP7 Genes [20].

**Ethical considerations:** the approval to carry out the research was obtained from the University of Kabianga Institutional Ethics Review Committee, Ethics Clearance Number: IERC/2020/005. The research permit was obtained from the National Commission of Science, Technology, and Innovation, Research License No: NACOSTI/P/21/9085. Permission was also obtained from the County commissioner of Kericho County, the Director of Education, and the Director of Health. Permission was also obtained from the Hospital's administration and Ethics committee. The study was explained to the participants before seeking written informed consent from them. Assistance was also rendered to the guardians who were unable to read. Participation was voluntary and respondents were liberal to withdraw at any stage of the study. Confidentiality was ensured by not having any sort of identification on the data collection tools.

## Results

**Sociodemographic characteristics of the participants and their guardians:** Table 1, a total of 200 children participated in the study. Of the 200 samples, 106 (53%) were from males whereas 94 (47%) were from females. The most affected age was the 21-30 months of age group at 26%. Age of 0 to 10 months-3/23 (13%), 11 to 20 months -3/23 (13%), 21 to 30 months -6/23 (26%), 31 to 40 months 5/23 (22%),41 to 50 months 3/23 (13%) and finally 51 to 60 months 3/23 (13%). The most affected demographics were children with parents or guardians who had secondary school education (51%). The most affected social economic status was the housewives (46.5%).

**Table 1:** demographic characteristics of children with acute gastroenteritis visiting Kericho County Referral Hospital, Kenya (N=200)

Characteristic		Frequency	Percent
**Gender**	Male	106	53
	Female	94	47
**Age (months)**	<8	7	3.5
	9-12	28	14
	13-18	11	5.5
	19 -23	6	3
	24-28	39	19.5
	29-33	14	7
	34-38	53	26.5
	39-43	9	4.5
	>43	33	16.5
**Parent/guardian education**	No formal education	10	5
	Primary	49	24.5
	Secondary	102	51
	Tertiary	39	19.5
**Parent/guardian occupation**	Self employed	34	17
	Farmer	34	17
	Formal employment	39	19.5
	Housewife	93	46.5

**Figure 1:**
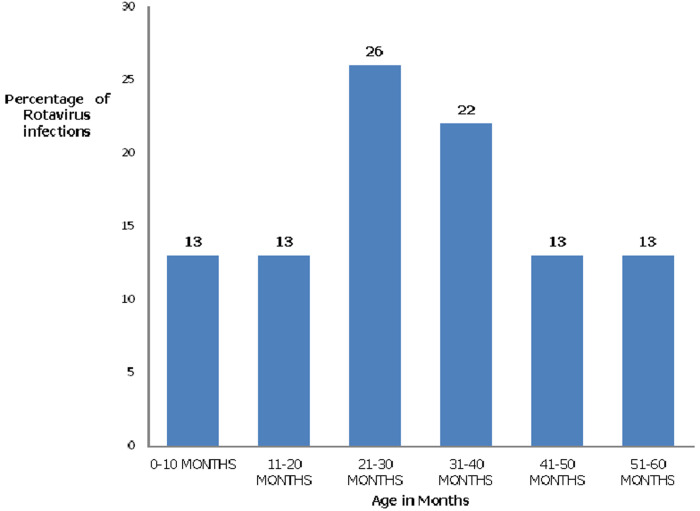
percentage of rotavirus infections in each age range

**Prevalence of rotavirus among the participants:** Rotavirus RNA electrophoretic patterns were observed in these samples as 11 segments. Twenty-two (96%) of the isolated strains were long strains except for one (4%) which did not exhibit the segments. The short electropherotypes were not found in the present study (Figure 1).

**Figure 2:**
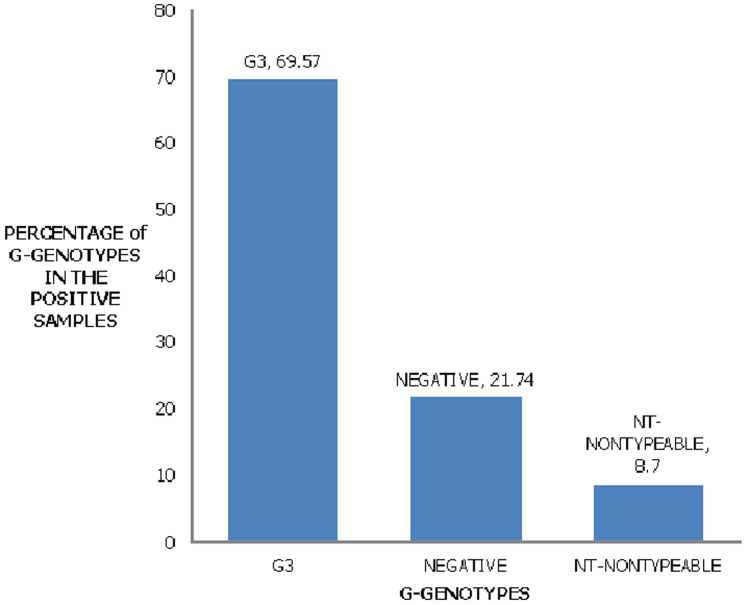
group A Rotavirus, G-genotypes identifying among children aged five years and below in Kericho County Referral Hospital

**Figure 3:**
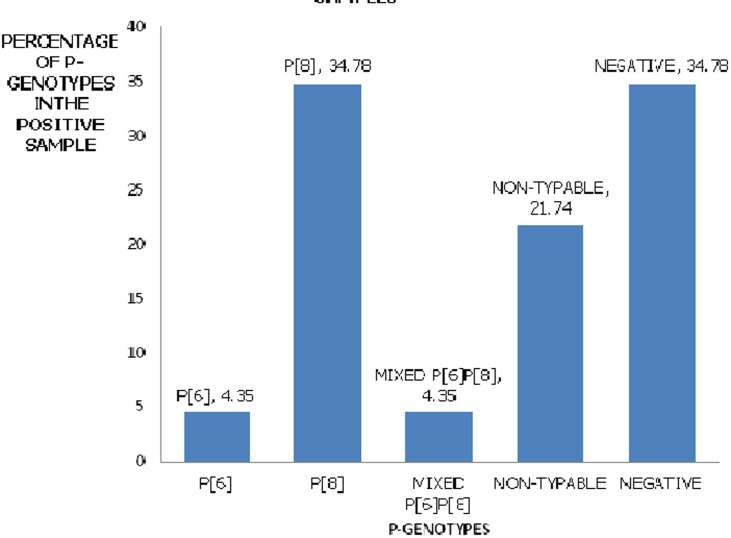
group A Rotavirus, P-Genotypes identifying among children aged five years and below in Kericho County Referral Hospital

**Genetic analysis of the rotavirus strains:** of the 23 positive samples of rotavirus, 16(69.57%) were G-type (VP7 associated), 2(8.7%) were non-typeable, and 5 (21.74%) were G-negative. When the G genotype was characterized using G1-G4, G6, G8, and G9 primers, only the G3 genotype was found (Figure 2). The P genotypes (VP4 associated) were found in 15/23 (65.22%) The P genotype was characterized using P[4], P[6], P[8], P[9], and P[10] specific primers. Of the 23 samples, the P genotypes present were as follows: P[6] was one (4.35%), Mixed P[6]P[8] was one (4.35%), P[8] was in eight samples (34.78%), non-typeable genotypes were in five samples (21.74%) and those negative for P genotype were eight (34.78%) (Figure 3).

**Combination of G and P types:** during the typing of assays it was also found that a specific G type would continuously be associated with a P-type; namely, G3, would coexist with P[8] that is G3P[8] which occurred in 8/23 (34.78%) and only once did a mixed infection occur of G3P[6]P[8] -1/23 (4.35%) (Table 2).

**Table 2:** G and P genotype combinations detected in children of five years and less in Kericho County Referral Hospital

G/P combination type	Children (N=23)(%)
**Common types**	
G3P[8]	8/23 (34.79%)
**Uncommon types**	
G3P[6]P[8]	1/23 (4.35%)
**Non typeable**	
GntPpos	3/23 (12.00%)
GposPnt	7/23 (30.43%)
GntPnt	4/23 (17.39%)

G and P combinations detected in children of 5 years and below; GntPpos- G non-typeable but with identified P type; GposPnt-P non-typeable but with identified G type. GntPnt- both G and P non-type able

**Limitations of the study:** the study had several limitations: it was difficult to make accurate conclusion due to results obtained during a relative short period of time and the small sample size. This scope was also limited due to lack of published data on rotavirus infections at Kericho County Referral Hospital. Moreover, only patients at the hospital setting were recruited in this study. In order to investigate the true burden of viral gastroenteritis, community-based studies which will include children in baby care centres, hospital based settings and larger health care catchment areas is necessary. Despite these limitations, this study provided the most comprehensive data to date on rotavirus characterization at Kericho County Referral Hospital.

## Discussion

The highest prevalence of infection was identified within the age group 21-30 months old children at 26%. The lowest prevalence was among children within the age group 2-4 months post-vaccination that is 0 to 10 months with vaccination taking place from 6 weeks. This different from the work done by C. Muendo *et al*. 2018 [14] where the highest infections were observed at the age group 17-24 months at 41.4% and J. Gikonyo *et al*. 2019 [10] who observed the highest infections at 13-24 months. This confirmed that the age of infection has shifted to the age group not covered by vaccination. This was also evident in the trends of infection observed in a study carried out before vaccine introduction that indicated the highest infections occurred at 6-17 months by Wanders EA *et al*. [21].

The prevalence rate of group A rotavirus in this study was 11.5% which is less compared to the overall rotavirus prevalence that was 14.5% in a study carried out at Kenyatta National Hospital in Nairobi [14]. This is also lower as compared to the prevalence of 15.2% obtained in a study in Nairobi County in 2019 [10]. This can be deduced that rotavirus vaccination is effective even in rural areas of counties deemed to be based more on agricultural backgrounds. This is an expected outcome witnessed immediately after vaccine introduction and studies done four- five years pre vaccine era. An example would be two studies published by Agutu *et al*. 2017 with 31.5% prevalence rate and 27.5% prevalence rate by Wandera EA *et al*. 2017 [22]. The current study confirms a decline in rotavirus prevalence in the study age group attributable to vaccine introduction in 2014.

The presence of a specific electropherotype pattern has long since been a diagnostic for the occurrence of individual Groups A, B, and C rotaviruses. Group A rotaviruses that tested positive through ELISA display a distinct pattern when subjected to PAGE and Silver staining. This confirmed that the positive samples were of group A rotaviruses. All samples presented 11 segments of dsRNA pattern similar to the positive Group A rotavirus controls that consisted of long and short strain samples with the exception of one which despite being positive in ELISA, did not give any result. There are two types of electropherotype patterns of group A rotavirus that are long and short electropherotypes. Long electropherotypes are linked to G1, G3, G4, and G9 whereas the short electropherotypes are mostly linked to G2. The characteristic grouping of rotavirus RNA segments of 4-2-3-2 was observed. This indicated that the samples were positive for Group A. It should be noted that the electropherotype pattern suggests but does not confirm a given genotype [23].

Studies on Rotavirus have been done in various parts of the world. It has been shown that G1 was the most predominant strain worldwide according to U. Desselberger 2014 and Seheri *et al*. 2018. This was followed by strains G2 -G4. In this study, the most predominant strain was G3 at a percentage of 69.57%. There were also combination infections such as G3P [8] at 34.79%, and G3P [6]P[8] at 4.35%. This is in line with the discoveries of Steele and Ivanoff, in 2003 that with each outbreak, new strains of rotavirus are found. Our findings are new compared to those by Kiulia *et al*. 2006 which indicated that the most predominant strain in the Meru North district in Kenya was G9. This also indicates a shift in the strains present from the ones that existed before vaccine introduction in Kenya to other strains post-vaccine introduction. In this case, the most prevalent in such areas such as Kiambu County Hospital was G1P[8] (28%), after that G9P[8] (12%), G8P[4] (7%), G1P[4] (5%), G9P[4] (4%), and G12P[6] (3%). In the yearly change of G and P genotypes, a major change from G9P[8] to G1P[8] was observed in 2012 [21]. On the contrary in this study, the most predominant were G3 strains thus indicating serotype placement or shift from the strains included in the vaccine to ones not used in the vaccine program.

These findings have implications, it is not clear whether the existing monovalent Rotarix vaccine that comprises of G1, P[8] live attenuated strain [24] or Rota Teq vaccine comprising of serotypes G1-G4 [24] will provide a cross-protection counter to emerging strains. Genotype P[4] and G3/G9P[8] combinations identified highlight the likelihood of rotaviruses going through re-assortment [25]. This is also evident with the study carried out in peri-urban regions in Central Kenya that observed a shift in the strains found among infected children, there was an increased detection of G2P[4], G3P[6] and G3P[8], which corresponded temporally with the scheduling of the vaccine introduction [22]. It should be noted that the vaccines in use might not be catering to the emerging strains.

## Conclusion

Gastroenteritis due to Rotavirus is one of the major causes of severe dehydrating diarrhea in children and is prevalent at Kericho County Referral Hospital. The G3 genotype was the main serotype affecting the children in the study and the most prevalent in circulation. Long electropherotypes were more than short electropherotypes. Epidemiologic observation of rotavirus is necessary for understanding the burden of rotavirus-caused gastroenteritis in Kericho County and its impact on the national health cost in Kenya. A variation in the genetic diversity of rotavirus strains was observed, there was a down surge in the prevalence of G1P[8] after vaccination and an increase in G2P[4] by 12.2% and G9P[8] by 20.4% contrary to what had been reported previously. This indicates that strains may be diversified due to climatic conditions, social economic factors, and even infrastructure among other factors. Arising from the findings, it is recommended that Rotavirus surveillance programs should be considered in the future, consideration of seasons considering climate and weather inconsistencies. Considering the observation of a shift of infection to older age groups, more surveillance is required to monitor the emerging strains. Due to the prevalence of the G3 strains which are not part of the makeup of the Rotarix vaccine, it is also advisable for the government to include other vaccines that may wholly cover the emerging strains of Rotavirus to increase the efficiency of the vaccination programs.

### What is known about this topic

- *Reviews of rotavirus prevalence in Kenya before vaccine introduction reveals a prevalence of 6% to 56% in children under five years of age;*
- *The most common strain of rotavirus among humans is rotavirus A*.

### What this study adds

- *This is the first study to determine the prevalence of rotavirus in Kericho County Referral Hospital which is based on a population that is largely dependent on Agriculture;*
- *The new rotavirus strain combinations existing within the population will assist in the type of vaccine that is being used*.
